# Comparing Depressive Symptoms, Emotional Exhaustion, and Sleep Disturbances in Self-Employed and Employed Workers: Application of Approximate Bayesian Measurement Invariance

**DOI:** 10.3389/fpsyg.2020.598303

**Published:** 2021-02-02

**Authors:** Louise E. Bergman, Claudia Bernhard-Oettel, Aleksandra Bujacz, Constanze Leineweber, Susanna Toivanen

**Affiliations:** ^1^Section for Work and Organizational Psychology, Department of Psychology, Stockholm University, Stockholm, Sweden; ^2^Division of Psychology, Department of Clinical Neuroscience, Karolinska Institutet, Solna, Sweden; ^3^Department of Psychology, Stress Research Institute, Stockholm University, Stockholm, Sweden; ^4^School of Health, Care and Social Welfare, Mälardalen University, Västerås, Sweden

**Keywords:** self-employed, Entrepreneurship, Sweden, emotional exhaustion, depressive symptoms, sleep disturbances, mental health problems, approximate measurement invariance

## Abstract

Studies investigating differences in mental health problems between self-employed and employed workers have provided contradictory results. Many of the studies utilized scales validated for employed workers, without collecting validity evidence for making comparisons with self-employed. The aim of this study was (1) to collect validity evidence for three different scales assessing depressive symptoms, emotional exhaustion, and sleep disturbances for employed workers, and combinators; and (2) to test if these groups differed. We first conducted approximate measurement invariance analysis and found that all scales were invariant at the scalar level. Self-employed workers had least mental health problems and employed workers had most, but differences were small. Though we found the scales invariant, we do not find them optimal for comparison of means. To be more precise in describing differences between groups, we recommend using clinical cut-offs or scales developed with the specific purpose of assessing mental health problems at work.

## Introduction

Do self-employed workers experience less depressive symptoms and emotional exhaustion than organizationally employed workers? Does the different types of work affect the quality of their sleep? Do those who are entirely self-employed and those who combine self-employment with organizational employment differ in their mental health problems? As self-employment, and self-employment in combination with organizational employment, is a reality for a growing proportion of workers, these are questions researchers recently have started to ask (Bureau and Dieuaide, 2018; Conen and Schippers, 2019; Torrès and Thurik, 2019). This new interest might come from the indications that mental health problems of self-employed workers seem to not only affect the individual worker but also business success and thus their ability to contribute to society (Stephan, 2018). Self-employed workers have essential and specific roles in our economy as they create jobs and contribute to innovation and economic productivity (Van Praag and Versloot, 2007; Cribb and Xu, 2020). Self-employed workers are sometimes also referred to as “entrepreneurs” to highlight the fact that they found and build businesses based on innovative ideas or own inventions. However, in here, we use the term “self-employed” to include virtually anybody who has registered an own business, no matter whether that is based on entrepreneurial spirit or necessity, a growing enterprise, or a small-scaled solo self-employment.

Studies indicate that self-employed workers value their mental health highly. They seem to make business decisions based on consequences for their mental health (Shepherd et al., 2009) and see good mental health as an indicator of their success (Wach et al., 2016). Also, deficient mental health is connected to job termination in self-employed workers (Hessels et al., 2020). Moreover, earlier research also indicate that mental health problems such as depressive symptoms and emotional exhaustion relate to self-employed workers’ intentions to leave self-employment (Wincent et al., 2008; Hessels et al., 2018). In sum, mental health does not only hold intrinsic value for the worker but is an important value for society as a whole.

Self-employment differs from organizational employment substantially, both when it comes to *who* is self-employed—e.g., differences in characteristics such as personality traits, age, gender, and education—and *how* they work—e.g., freedom to plan one’s work, certainty of income, and social context. In some cases, workers are both employed by an organization and self-employed, making up a group of workers who combine these two substantially differing work settings (henceforth called *combinators*; in some research called hybrid entrepreneurs). Thus, to understand how these types of work affect mental health, it is essential to study mental health problems of self-employed workers and combinators separately from the employed workers.

Studies comparing mental health of employed workers and self-employed workers or combinators yield contradictive findings. One reason for this could be the utilization of scales developed for the general population and commonly used to describe mental health problems in employed workers, without collecting validity evidence for the scale’s new purpose: comparing groups of workers with quite different employment circumstances. Thus, in this paper, we test whether employed workers, self-employed workers and those who combine these types of work are comparable regarding three common indicators of mental health problems: *depressive symptoms*, *emotional exhaustion*, and *sleep disturbances*.

### Self-Employed Workers, Combinators, and the Organization of Their Work

Two main aspects affect both mental health of self-employed and employed workers, and their interpretation of assessment scales: who they are and how they work. Self-employed workers are more often male, are older, and are less educated than the average employed worker is (Leineweber et al., 2016; Bernhard-Oettel et al., 2019). Further, people with certain characteristics are more frequently self-employed, for example, those with higher self-efficacy, need for achievement (Frese and Gielnik, 2014), and psychological capital (Baron et al., 2016). With regard to combinators, they seem to be more highly educated and possess more skills, knowledge, and experience than the general workers, and are more often active in knowledge-intensive and innovative industries (Folta et al., 2010; Petrova, 2012). Some of them have the intention to leave organizational employment and become full time self-employed, while others are content with combining employment and self-employment (Thorgren et al., 2016; Solesvik, 2017). Thus, employed workers, self-employed workers, and combinators differ substantially with regards variables that are also related to differences in health.

Employed workers, self-employed workers, and combinators also differ in how they work. Self-employed workers engage in an activity for their own account and are able to recruit their own employees (European Union Foundation, 2009). This gives them a distinctive freedom to organize their work as they want to (Obschonka and Silbereisen, 2015). This freedom affects all aspects of work: choice of tasks, time schedule, and utilization and development of one’s skills (Financial Stability Financial Services and Capital Markets Union, 2018). Degree of freedom to organize one’s work has been shown to decrease stress levels and increase health in both employed and self-employed workers (Theorell et al., 2015; Aronsson et al., 2017; Bujacz et al., 2017; Madsen et al., 2017; Stephan et al., 2020b). However, these positive aspects related to freedom do not paint the whole picture.

While self-employment is often described positively with respect to the organization of work and its effects on mental health, reality may not be that simple (Stephan, 2018). With the freedom following self-employment, other difficulties arise such as possible restrictions to income, lack of boundaries between work and life, and lack of peer support and responsibility for employees (Stephan, 2018). Too much freedom and lack of clear boundaries between work and leisure time might affect mental health negatively (Kubicek et al., 2014). Further, other potential difficulties may relate to the lack of income stability, insecure future, strong dependence on one’s own health, and weak insurance security (Grant and Ferris, 2012). For combinators, the working conditions of both employed and self-employed workers are present, and on top of that, they need to find a way to balance their two (or more) jobs. Difficulties might arise in the form of day-to-day balancing of time and resources (Murgia and Pulignano, 2019). Hence, the different groups of workers vary in the organization of their work and the benefits and challenges affecting their health.

### Work Stress and Mental Health Problems of Employed Workers, Self-Employed Workers, and Combinators

The job-demand control model is the most popularly used model to theoretically understand and explain how differences in the organization of work may evoke differences in health, for example, in depressive symptoms and emotional exhaustion (Häusser et al., 2010). The model holds that too high demands, too difficult or many tasks can be negative for health, but high control, many possibilities, and autonomy is positive for health. The job demand-control model has also been used to compare organizationally employed with self-employed workers, and researchers have found that self-employed workers have higher job control (and thus higher autonomy which is beneficial for health) than employed workers (Stephan and Roesler, 2010; Bernhard-Oettel et al., 2019). The link between work organization and sleep disturbances may better be explained with the stressor-detachment theory. In this model, job stressors do not lead to a problem as long as there is enough recovery. If job stressors are highly present and there is no room for recovery (such as sleep), the worker will experience psychological detachment and impaired health (Sonnentag and Fritz, 2015). This model may be particularly relevant for self-employed workers and combinators, as the lack of boundaries between work and life and longer hours potentially affect recovery and thus, mental health.

Looking at the empirical evidence regarding differences in mental health problems between self-employed workers, employed workers and combinators, studies are contradictory. Some studies report no meaningful differences between self-employed and employed workers when they compared mental health problems on a general level (Prottas and Thompson, 2006; Andersson, 2008; Tuttle and Garr, 2009), or that they are restricted to the beginning of self-employment (Stephan et al., 2020a). However, when focusing on more specific mental health indicators such as depressive symptoms, emotional exhaustion, and sleep disturbances, results were more diverse. While Jamal (2007) found that self-employed workers are more exhausted than employed workers, Sikora and Saha (2009) found the opposite. Rietveld et al. (2015) found that self-employed workers have less depressive symptoms than employed workers, but Parslow et al. (2004) found no differences. Maeda et al. (2019) found that while sleep disturbances were present in self-employed but not in employed women, the opposite was true for men. These contradictory results make evident that it might be beneficial to study different types of mental health problems separately and to scrutinize the scales utilized.

### Validity Evidence of Mental Health Problem Scales

An important reason for understanding the contradictions in differences in mental health problems between groups of workers is that many studies do not test for differences in how different workers apprehend the assessment tools. Researchers must collect validity evidence to assure that the scales they use to assess a construct do so accurately and comparably in all groups of workers under study. As stated in *Standards for educational and psychological testing* (American Educational Research Association et al., 2014), validity evidence is not collected for a specific assessment scale, but for a specific purpose, and a specific group of respondents. This implies that researchers need to collect new validity evidence every time the purpose or context of the scale alters. Researchers use scales originally developed to describe the general population to compare groups of workers with little heed to the substantial differences in purpose. Transferring these scales without first investigating their aptness for this new purpose might lead to misinterpretations of results. Comparisons between employed workers, self-employed workers, and combinators might just show differences in how the scales are interpreted by the three groups, not real differences in mental health. For example, self-employed workers who are single-handedly responsible for their business may often worry or feel tired, without it affecting their engagement and interest. Whereas for organizationally employed workers, feelings of tiredness, and low energy and interest may have stronger associations and reflect aspects of mental exhaustion or depressive symptoms with more congruence.

### Study Aims and Research Questions

Employed workers, self-employed workers, and combinators differ substantially as groups, in both personal characteristics, and in organization of their work. Studies comparing mental health problems in these groups have yielded contradictory findings, which might be due to the lack of validity evidence for utilization of the scales for comparison of these groups of workers. Moreover, many of the earlier studies have overlooked the specific group of combinators. In part, this may also have contributed to the inconclusive findings. Accordingly, in this study we are guided by the following research questions:

Q1. Are scales assessing mental health problems including depressive symptoms, emotional exhaustion, and sleep disturbances measurement invariant in employed workers, self-employed workers, and combinators?

Q2. Do employed workers, self-employed workers, and combinators differ in degree of depressive symptoms, emotional exhaustion, and sleep disturbances?

## Materials and Methods

### Data Collection and Respondents

Since 2006, Statistics Sweden (SCB), on behalf of the Stress Research Institute, collect data for the Swedish Longitudinal Occupational Survey of Health (SLOSH), a national representative cohort study (Magnusson Hanson et al., 2018). SLOSH is a follow-up of the participants of the Swedish Work Environment Surveys (SWES) and comprises today all SWES participants 2003–2011 (*n* = 40,877). As SLOSH is based on the SWES, it can be regarded as approximately representative of the Swedish working labor market. All labor market sectors and occupations are represented, and the number of men and women is approximately equal. SLOSH is conducted every second year by means of a pen-and-paper questionnaire in two versions; one for respondents who work at least 30% (which in Sweden generally is 12 h per week) and one for those who have left the working force, either permanently or temporarily. The current study is based on participants who responded to the fifth wave of SLOSH conducted in 2014 (response rate 53%) and who were either employed workers (*n* = 14,232), self-employed workers (*n* = 1034), or combinators (*n* = 339). The mean age of respondents was 51 (SD = 10) years, ranging from 20 to 76 years. The study sample included more women (57%) than men (43%). The majority of the participants were born in Sweden (94%), married of cohabitating (79%), and half of the participants had no children living at home (54%). Most participants had either a university education 3 years or longer (30%), or 3 or 4 yearlong high school education (24%). The remaining ones had either 2 years of high schooling or training school (22%), went to university for less than 3 years (15%), or had elementary school education (10%).

The samples of workers are unequal in size and that is a problem for comparative analyses. We therefore chose to match sample sizes before comparisons (see Table 1). We created these subsamples by randomly drawing subsamples of the original data from organizational employees, or self-employees, respectively. Of all randomly generated subsamples, we chose the ones with closest match to the original data considering important background variables (gender, civil status, children, socioeconomic status, education level, and region of birth). Therefore, the sample description as given earlier is still valid for the smaller subsamples; the exact distribution of background variables in each subsample can be seen in the Supplementary Material. We based comparisons between self-employed workers (called “SE large sample”) and employed workers (called “EM large sample”) on a sample of *N* = 1034 in each group; see Table 1). We based comparisons between employed workers and combinators, and self-employed workers and combinators, on subsamples of 339 participants in each group (named “OE small sample,” “SE small sample,” and “CO small sample”). Even in these smaller samples, the sample sizes are still adequate for the analysis, as we employed simple one-factor models only (for more information on sample sizes in SEM analysis, see Wolf et al., 2013).

**TABLE 1:** Subsamples.

	**Employed workers**	**Self-employed workers**	**Combinators**	**Comparison model for MI test**
Sample size *n* = 1,034	Em large sample	Se large sample	–	Emse
Sample size *n* = 339	Em small sample	Se small sample	Co small sample	Emco, Seco

*Em, employed workers; Se, self-employed workers; and CO, combinators.*

In line with Groves (2006) recommendation, we tested for response rate bias using those who answered the questionnaire after having received a reminder as a proxy for non-respondents. First-wave respondents had slightly better results on all three scales (depressive symptoms: Δ = -0.09, 95% CI = -0.12, -0.06, emotional exhaustion: Δ = -0.02, 95% CI = -0.20, -0.10, and sleep disturbances: Δ = 0.002, 95% CI = -0.04, 0.04). The 95% CI did not overlap for depressive symptoms and emotional exhaustion, but for all three constructs, the differences are small and thus response rate bias is small.

### Assessment Scales

All item and construct means, standard deviations, and correlation may can be viewed in Table 2. We assessed *depressive symptoms* with the symptom checklist-core depression (SCL-CD_6_) including six items (see Table 3; Magnusson Hanson et al., 2014b). The researchers developed SCL-CD_6_ to assess and describe occurrence of depressive symptoms, major depression, and changes over time in different populations. SCL-CD_6_ uses a small number of depression core characteristics necessary for diagnosis so that the scores sum up to a meaningful severity assessment (Magnusson Hanson et al., 2014b). The respondents rate the items on a 5-point answer format from 1 *Not at all* to 5 *A lot*.

**TABLE 2:** Means, SDs, and correlations for all items and indexes for the full sample (*N* = 15,605; all correlations: *p* = 0.000).

	**Mean**	**SD**	**Correlations**																	
			**1**	**2**	**3**	**4**	**5**	**6**	**SCL-CD_6_**	**7**	**8**	**9**	**10**	**11**	**12**	**SMBQ**	**13**	**14**	**15**	**16**
1. SCL-CD_6_1	2.189	1.067	1																	
2. SCL-CD_6_2	1.700	0.958	0.630	1																
3. SCL-CD_6_3	1.712	0.962	0.469	0.616	1															
4. SCL-CD_6_4	2.037	1.089	0.525	0.629	0.699	1														
5. SCL-CD_6_5	1.793	0.967	0.582	0.638	0.545	0.555	1													
6. SCL-CD_6_6	1.827	1.035	0.659	0.657	0.574	0.636	0.697	1												
Depressive symptoms	1.876	0.831																		
7. SMBQ1	3.043	1.574	0.634	0.479	0.389	0.438	0.480	0.552	0.607	1										
8. SMBQ2	2.370	1.570	0.479	0.506	0.430	0.465	0.494	0.549	0.595	0.497	1									
9. SMBQ3	2.535	1.596	0.595	0.542	0.448	0.489	0.525	0.620	0.656	0.625	0.750	1								
10. SMBQ4	1.892	1.419	0.510	0.538	0.433	0.472	0.500	0.577	0.617	0.527	0.662	0.764	1							
11. SMBQ5	2.643	1.633	0.601	0.577	0.479	0.537	0.537	0.632	0.685	0.618	0.671	0.794	0.746	1						
12.SMBQ6	2.071	1.489	0.481	0.424	0.351	0.374	0.422	0.473	0.514	0.529	0.473	0.541	0.523	0.559	1					
Emotional exhaustion	2.423	1.279							0.743											
13. KSQ1	2.426	1.194	0.346	0.332	0.275	0.332	0.297	0.325	0.389	0.340	0.282	0.322	0.324	0.334	0.302	0.384	1			
14. KSQ2	2.626	1.296	0.368	0.322	0.283	0.353	0.290	0.335	0.398	0.365	0.301	0.340	0.328	0.344	0.251	0.390	0.559	1		
15. KSQ3	2.580	1.262	0.365	0.312	0.273	0.329	0.294	0.329	0.389	0.390	0.288	0.330	0.311	0.333	0.259	0.386	0.391	0.608	1	
16. KSQ4	2.761	1.316	0.440	0.362	0.315	0.386	0.335	0.381	0.453	0.442	0.320	0.381	0.350	0.390	0.315	0.444	0.493	0.688	0.586	1
Sleep disturbances	2.597	1.035							0.499							0.492				

**TABLE 3:** Survey items.

*How much during the last week have you been troubled by:*
SCL-CD_6_ 1 Lethargy or low in energy?
SCL-CD_6_ 2 Feeling blue?
SCL-CD_6_ 3 Blaming yourself?
SCL-CD_6_ 4 Worrying too much?
SCL-CD_6_ 5 Feeling no interests in things?
SCL-CD_6_ 6 Everything is an effort?
*Below we describe a number of states that every one of us can experience now and then. Please fill in to what degree these you experience these states during a major part of your day.*
SMBQ1 I feel tired.
SMBQ2 I feel “fed-up”.
SMBQ3 My “batteries” are “empty”.
SMBQ4 I feel burned out.
SMBQ5 I feel mentally fatigued.
SMBQ6 I feel no energy for going to work in the morning.
*How often have you been troubled by the following in the last 3 months?*
KSQ1 Difficulties falling asleep.
KSQ2 Repeated awakenings with difficulties falling asleep.
KSQ3 Premature (final) awakening.
KSQ4 Disturbed/restless sleep.

We used the revised subscale for emotional exhaustion and fatigue from the Shirom Melamed Burnout Questionnaire (SMBQ) to assess *emotional exhaustion* (see Table 3; Shirom, 1989; Melamed et al., 1992; Shirom et al., 1997; Shirom and Melamed, 2006). Validity evidence for SMBQ have been collected over a broad set of populations and purposes including clinical, military, and different working populations but not in self-employed specifically. In the current study, we include six items (see Table 3) deemed a sufficient scale for describing emotional exhaustion in the general population (Shirom and Melamed, 2006). Respondents rated these items on a 6-point answer format from 1 *Few times or never* to 6 *Every day*.

*Sleep disturbances* relate to difficulties falling asleep, restless sleep, and premature awakening and have been found to be universal indicators of mental health problems (Nordin et al., 2013). We used a subscale (see Table 3) assessing sleep disturbances from the Karolinska Sleep Questionnaire (KSQ; Nordin et al., 2013; Magnusson Hanson et al., 2014a, 2017). KSQ was developed to describe subjective sleep and sleepiness in a general population (Kecklund and Åkerstedt, 1992). Respondents rated the four items on a 6-point answer format from 1 *Few times or never* to 6 *Always/Five times a week or more*.

### Analysis Strategy

We started out testing the structural relationships of each construct (depressive symptoms, emotional exhaustion, and sleep disturbances) in each subsample by using confirmatory factor analysis (CFA) with Bayesian structural equation modeling (BSEM; input files 1–25, Supplementary Material). BSEM provides more accurate results for data with asymmetric distributions, as it does not assume that normal distributions underlie the parameters of the model (van de Schoot et al., 2011). With BSEM, one can define a more realistic model, closer to the real-life phenomena one wants to assess. This is possible by the use of priors: information that is fed into the model based on prior studies (hence the name; Van de Schoot et al., 2014 provide a thorough introduction to Bayesian statistics). Here, we use priors to relax constraints that are usually set to zero, so that the model was tested allowing small variation among groups (Muthén and Asparouhov, 2012; Asparouhov and Muthén, 2019). When fitting the CFAs, we used priors to allow small residual covariances to model influences unrelated to the factors (e.g., wording effects and context; Asparouhov et al., 2015). In fitting these models, we followed the process described by Asparouhov et al. (2015). We found that this process yielded unnecessarily influential priors, and adjusted them so that the posterior predictive *p* value (PPP) value was just above 0.05 (Asparouhov et al., 2015), and the CI for the observed and the replicated χ^2^ values included zero, thus indicating as uninfluential priors as possible.

To answer the first research question, we conducted a series of multi-group CFAs to test the approximate measurement invariance (MI) of the three constructs (depressive symptoms, emotional exhaustion, and sleep disturbances) between the three groups (input files 26–52, Supplementary Material). We employed a stepwise procedure, beginning with evaluating the least restrictive constraints (Brown, 2015). Hence, we first tested configural MI (does the measurement model apply in all groups?), followed by tests for metric MI (are the factor loadings invariant across groups?), and finally, tests for scalar MI (are the factor loadings and intercepts invariant across groups?). To identify the model, we fixed the factor variance to one in one group only and in the other group, we placed the equality constraints on the factor loadings while we estimated a factor variance (Yoon and Millsap, 2007). This method minimizes problems caused by commonly used solutions such as constraining the first factor loading to one (Bauer and Hussong, 2009). Taking advantage of the benefits of approximate MI (a full description of this process can be found in Muthén and Asparouhov, 2012), we used informative priors to model small differences between groups on the factor loadings (in the metric and scalar model) and intercepts (for the scalar model). Such differences may occur due to sampling, wording, and interpretation issues, which are not substantially important (Van De Schoot et al., 2013). We set these priors to 0.01 to allow for non-relevant differences between groups. For each construct, we tested three models on each level (configural, metric, and scalar) and ran the following set of comparative analyses: (1) employed workers to self-employed workers, “Emse” model; (2) employed workers to combinators, “Emco” model; and (3) self-employed workers to combinators, “Seco” model.

We used the PPP and CIs for the observed and the replicated χ^2^ values to decide whether a model had good fit and to compare models. Muthén and Asparouhov (2012) suggest that a model has good fit when PPP is above zero and CI for the observed and the replicated χ^2^ values include zero. In addition, we used deviance information criteria (DIC) and Bayesian information criteria (BIC) to compare models, where models with lower DIC and BIC have better fit (McElreath, 2020). Lastly, we used a number of model fit indicators for a comprehensive evaluation of model fit. More specifically, we inspected the root mean square error of approximation (RMSEA; below 0.05 was deemed good, 0.08 acceptable) and comparative fit index (CFI; above 0.95 was deemed good) and Tucker–Lewis index (TLI; above 0.95 was deemed good; Kelloway, 2015). Importantly, we used these values as guidelines and not dichotomous cut-off points because of the problems this entitle, taking in background evidence, study design, data quality, and our understanding of underlying mechanisms into consideration (Gelman and Stern, 2006; Cohen, 2016; Amrhein et al., 2019).

In all analyses earlier, we performed model estimation with maximum 2,000,000 and minimum 10,000 iterations. We used Markov chain Monte Carlo (MCMC) Gibs sampling in Mplus version 8. We used the WAMBS checklist (When to worry and how to Avoid the Misuse of Bayesian Statistics) by Depaoli and Van de Schoot (2017). The purpose of this checklist is to improve transparency and replication, and with it, we checked our results to test that the results were not consequences of statistical artifacts.

To decide whether the groups differ in depressive symptoms, emotional exhaustion, and sleep disturbances (research question 2), we made two comparisons. First, we compared the latent factor means in the fully invariant (scalar) model, using the *p* values and 95% CI to estimate the probability that there is a true mean difference not equal to zero. Second, we compared an observed index variable for the constructs, calculating *t*-test to estimate the probability that there is a true mean difference not equal to zero. For the index variables, we also calculated Cohen’s D to better understand the magnitude of mean differences. We conducted these analyses in R (R Core Team, 2019).

## Results

### BSEM CFA With Identification of Small Priors for Residual Covariances

In the initial CFAs, we found small residual covariances for the models of depressive symptoms and emotional exhaustion. Exact priors of all models can be found in the input files in the Supplementary Material. For the sleep disturbances construct, we did not need to define priors, as fit was adequate without them in all three subsamples.

### BSEM Multi-Group CFA Test of Approximate Measurement Invariance

We present the results of the approximate MI test of *depressive symptoms* in Table 4. For the configural models the PPP values (all above zero), RMSEA (all reach at least acceptable fit of 0.08), CFI, and TLI (above 0.95) indicated good fit, while the 95% CI difference for the observed and the replicated χ^2^ values did not include zero. As most indices indicated acceptable or good fit, and the 95% CI difference for the observed and the replicated χ^2^ values were close to the criteria, fit was acceptable. The metric models generally fitted slightly better with regard to all indices except from BIC, which got worse. The CFI and PPP value remained unchanged with the exception of the Seco model for which the PPP value slightly increased. In sum, the metric models had generally better fit than the configural models. Scalar invariance tests yielded contradictory findings. For the Emse and Emco models, 95% CI difference for the observed and the replicated χ^2^ values, PPP, DIC, and TLI indicated slightly improved model fit, CFI remained unchanged, but BIC and RMSEA indicated slightly worse fit. For the scalar Seco model, RMSEA indicated better fit, CFI and TLI remained unchanged, and the remaining indicators showed worse fit than for metric invariance. For example, there were deteriorations in DIC and BIC (between 0.6 and 8.6 points), and RMSEA (between 0.002 and 0.004 points). However, all these changes were very small indicating no substantial deterioration of fit for any of the models through the increasing strictness. Given these small differences, we accepted the scalar model for all three groups.

**TABLE 4:** Measurement invariance analyses: Depressive symptoms.

**MI**	95% CI diff.*	**PPP**	**DIC**	**BIC**	**RMSEA (90% CI)**	**CFI (90% CI)**	**TLI (90% CI)**
***Employed and Self-employed workers (Emse model)***							
Configural	2.786–71.453	0.017	27,437.210	27,853.120	0.057 (0.037–0.075)	0.995 (0.992–0.998)	0.986 (0.977–0.994)
Metric	2.599–70.527	0.017	27,436.257	27,860.702	0.056 (0.036–0.073)	0.995 (0.992–0.998)	0.987 (0.978–0.995)
Scalar	2.029–70.251	0.019	27,435.446	27,868.630	0.054 (0.035–0.070)	0.995 (0.992–0.998)	0.988 (0.979–0.995)
***Employed workers and combinators (Emco model)***							
Configural	4.080–60.658	0.013	9,493.544	9,862.208	0.064 (0.047–0.080)	0.986 (0.978–0.992)	0.982 (0.972–0.990)
Metric	4.020–60.613	0.013	9,492.424	9,868.716	0.063 (0.046–0.078)	0.986 (0.978–0.992	0.982 (0.973–0.991)
Scalar	2.530–58.255	0.017	9,489.309	9,875.656	0.059 (0.043–0.074)	0.986 (0.979–0.993)	0.984 (0.976–0.992)
***Self-employed workers and combinators (Seco model)***							
Configural	3.225–64.714	0.015	9,421.861	9,781.189	0.073 (0.050–0.092)	0.985 (0.976–0.993)	0.976 (0.961–0.988)
Metric	2.001–63.210	0.018	9,419.332	9,787.758	0.069 (0.047–0.088)	0.985 (0.976–0.993)	0.978 (0.965–0.990)
Scalar	2.550–63.956	0.017	9,419.970	9,794.199	0.070 (0.048–0.088)	0.985 (0.976–0.993)	0.978 (0.964–0.990)

*95% CI for the difference between observed and replicated χ^2^ values.

We present the results of the BSEM multi-group CFAs of *emotional exhaustion* in Table 5. Overall, fit indices reached the criteria for acceptable model fit for the configural models. The metric models mostly fitted better on all indices, with a few exceptions, as BIC, CFI, and PPP value in the Emse model was unchanged. Hence, we deemed the stricter metric model as having good enough fit to continue to test scalar invariance. The results show that CFI and TLI values remained unchanged and indicated good fit in all models, PPP remained above zero for all models, and RMSEA indicated good fit for the Emse model, and acceptable fit in the other two models. DIC decreased slightly, whereas BIC increased with 7–8 points. Finally, we noted a minor deterioration in 95% CI difference for the observed and the replicated χ^2^ values in the Emse model, whereas this difference included zero for the Emco model. Altogether, these results indicated that the scalar model fit did not differ substantially from the metric model, and hence, we accepted the scalar model for all three groups.

**TABLE 5:** Measurement invariance analyses: Emotional exhaustion.

**MI**	95% CI diff.*	**PPP**	**DIC**	**BIC**	**RMSEA (90% CI)**	**CFI (90% CI)**	**TLI (90% CI)**
***Employed and self-employed workers (Emse model)***							
Configural	0.705–66.444	0.023	36,496.642	36,916.884	0.050 (0.031–0.065)	0.996 (0.993–0.998	0.991 (0.985–0.996)
Metric	0.553–65.579	0.023	36,495.548	36,924.887	0.048 (0.030–0.063)	0.996 (0.993–0.998)	0.992 (0.986–0.997)
Scalar	1.041–66.541	0.022	36,495.355	36,933.628	0.048 (0.030–0.062)	0.996 (0.993–0.998)	0.992 (0.986–0.997)
***Employed workers and combinators (Emco model)***							
Configural	1.135–58.120	0.021	12,502.980	12,871.131	0.062 (0.043–0.078)	0.988 (0.980–0.994)	0.984 (0.975–0.992)
Metric	0.407–56.471	0.024	12,499.761	12,878.243	0.058 (0.040–0.073)	0.988 (0.981–0.994)	0.986 (0.978–0.993)
Scalar	−0.347 to 55.441	0.026	12,497.372	12,885.827	0.055 (0.038–0.070)	0.988 (0.981–0.994)	0.987 (0.979–0.994)
***Self-employed workers and combinators (Seco model)***							
Configural	2.256–57.960	0.017	12,331.436	12,701.710	0.061 (0.044–0.076)	0.988 (0.981–0.994)	0.985 (0.977–0.992)
Metric	1.328–56.751	0.020	12,328.993	12,709.288	0.058 (0.042–0.072)	0.988 (0.981–0.994)	0.986 (0.979–0.993)
Scalar	1.297–56.101	0.020	12,326.705	12,717.056	0.056 (0.040–0.070)	0.988 (0.982–0.994)	0.987 (0.980–0.994)

*95% CI for the difference between observed and replicated χ^2^ values.

We present the results of the BSEM multi-group CFAs of *sleep disturbances* in Table 6. In the configural models, all indices indicated good fit, with the exception of RMSEA, which indicated acceptable fit for the Emse and Emco models, and not acceptable fit in the Seco model. The metric models generally had slightly better fit on all indices, except from BIC, which increased with 15.5 (Emse), 6.6 (Emco), and 7.0 (Seco). While RMSEA improved for the Seco model, it still did not fully reach the criteria of acceptable fit (0.089). Apart from BIC and RMSEA, the metric models had good fit, and we continued to the scalar models. Here, the models generally increased even more in fit with regard to all indices, except for BIC, which was unchanged (Emse), or increased with 7.0 (Emco) and 6.7 (Seco). For the Seco model, RMSEA now had acceptable fit. Because of the good fit of all models and general improvement observed with increasing model strictness, we accepted the scalar model for all three groups.

**TABLE 6:** Measurement invariance analyses: Sleep disturbances.

**MI**	95% CI diff.*	**PPP**	**DIC**	**BIC**	**RMSEA (90% CI)**	**CFI (90% CI)**	**TLI (90% CI)**
***Employed and self-employed workers (Emse model)***							
Configural	−2.723 to 37.764	0.041	23,573.264	23,707.988	0.065 (0.043–0.086)	0.994 (0.990–0.998)	0.983 (0.971–0.993)
Metric	−3.117 to 37.312	0.046	23,572.170	23,715.703	0.059 (0.039–0.079)	0.995 (0.990–0.998)	0.986 (0.975–0.994)
Scalar	−2.812 to 36.978	0.045	23,571.583	23,723.475	0.057 (0.037–0.076)	0.995 (0.990–0.998)	0.987 (0.977–0.994)
***Employed workers and combinators (Emco model)***							
Configural	−13.198 to 27.546	0.237	7,746.633	7,854.963	0.069 (0.000–0.119)	0.993 (0.980–1.000)	0.980 (0.941–1.000)
Metric	−14.163 to 25.927	0.265	7,744.403	7,861.602	0.056 (0.000–0.101)	0.994 (0.981–1.000)	0.987 (0.958–1.000)
Scalar	−14.420 to 25.339	0.275	7,743.033	7,868.603	0.049 (0.000–0.092)	0.995 (0.982–1.000)	0.990 (0.965–1.000)
***Self-employed workers and combinators (Seco model)***							
Configural	−4.664 to 36.124	0.063	7,770.467	7,878.770	0.104 (0.063–0.143)	0.984 (0.969–0.994)	0.953 (0.911–0.983)
Metric	−5.386 to 35.110	0.069	7,768.568	7,885.762	0.089 (0.053–0.123)	0.984 (0.970–0.994)	0.965 (0.934–0.988)
Scalar	−5.738 to 33.867	0.077	7,766.826	7,892.437	0.080 (0.047–0.111)	0.985 (0.971–0.995)	0.972 (0.946–0.991)

*95% CI for the difference between observed and replicated χ^2^ values.

### Comparison of Groups With *t*-Tests

**FIGURE 1:**
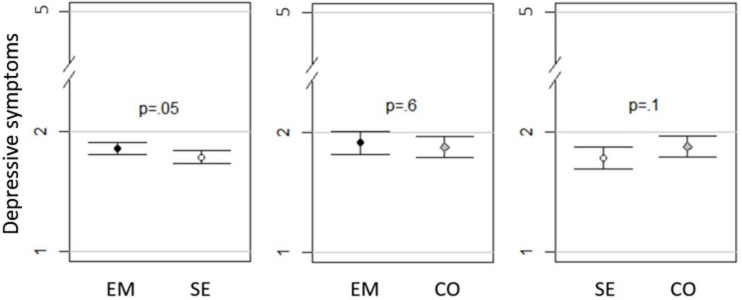
Mean depressive symptoms with 95% Cl and *p*-values in employed (EM), self-employed workers (SE), and combinators (CO).

**FIGURE 2:**
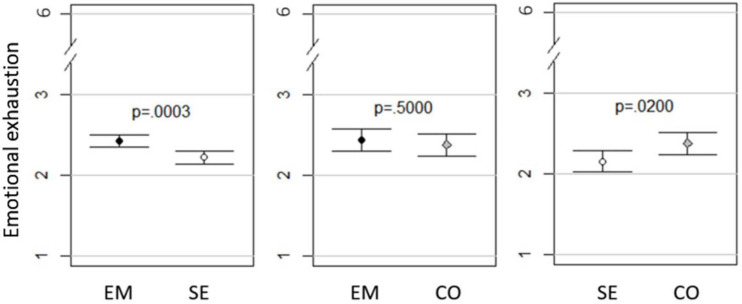
Mean emotional exhaustion with 95% Cl and *p*-values in employed (EM), self-employed workers (SE), and combinators (CO).

**FIGURE 3:**
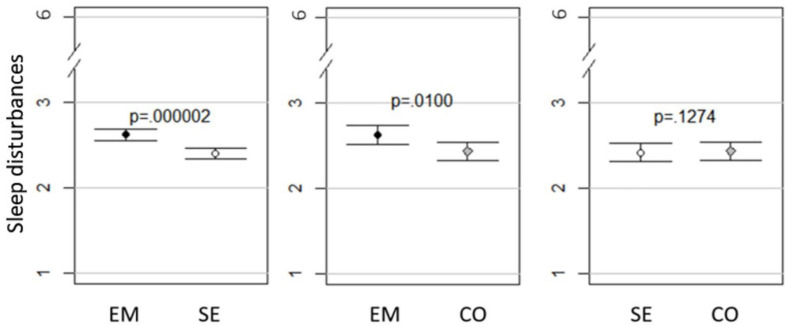
Mean sleep disturbances with 95% Cl and *p*-values in employed (EM), self-employed workers (SE), and combinators (CO).

As presented in Figures 1–3 and Table 7, small to minor mean differences existed between all three groups for depressive symptoms, emotional exhaustion, and sleep disturbances. As depicted in Figure 1, self-employed workers experienced less *depressive symptoms* than combinators, who in turn experienced less than employed workers did. Of these differences, only the difference between self-employed workers and employed workers had high probability of mean differences not equal to zero (*p* = 0.05). This difference was small, as indicated by Δ mean of the observed scale (0.074 on the 5-point scale) and Cohen’s D (0.056). The same pattern was present in *emotional exhaustion*, which is presented in Figure 2. Both the differences between self-employed workers and employed workers (*p* = 0.003), and self-employed workers and combinators (*p* = 0.02) had high probability of mean differences not being equal to zero. Looking at Δ mean of the observed scale and Cohen’s D, the differences were small for both the Emse comparison (0.207 on the 6-point scale and Cohen’s D 0.129) and the Seco (-0.220 on the 6-point scale and Cohen’s D 0.139). For sleep disturbances, presented in Figure 3, self-employed workers reported least disturbances, then combinators and employed workers the most. Of these differences, the Emse (*p* = 0.000002) and Emco (*p* = 0.01) differences had high probability of mean differences not being equal to zero and Seco medium probability of mean differences not being equal to zero (*p* = 0.1274). As for the other constructs, these differences were small for Emse (0.217 on the 6-point scale and Cohen’s D 0.152), Emco (0.190 on the 6-point scale and Cohen’s D 0.135), and Seco (-0.021 on the 6-point scale and Cohen’s D 0.015).

**TABLE 7:** Depressive symptoms, emotional exhaustion, and sleep disturbances in employed (EM) and self-employed workers (SE) and combinators (CO).

	****Δ** Mean**	**SD**	**Lower CI 2.5%**	**Upper CI 2.5%**	***p* value**	**Cohen’s D**
***Depressive symptoms***						
Emse 1	0.094	0.071	–0.235	0.044	0.090	
Emse 2	0.074	0.037	–0.001	0.146	0.050	
						0.056
Emco 1	0.052	0.091	–0.230	0.126	0.284	
Emco 2	0.038	0.066	–0.091	0.168	0.600	
						0.029
Seco 1	–0.107	0.177	–0.233	0.465	0.262	
Seco 2	–0.096	0.064	–0.221	0.030	0.10	
						0.074
***Emotional exhaustion***						
Emse 1	0.154	0.057	–0.267	–0.044	0.003	
Emse 2	0.207	0.057	0.094	0.319	0.001	
						0.129
Emco 1	0.032	0.089	–0.206	0.142	0.359	
Emco 2	0.064	0.097	–0.126	0.255	0.500	
						0.041
Seco 1	–0.185	0.090	0.009	0.364	0.019	
Seco 2	–0.220	0.096	–0.409	–0.030	0.020	
						0.139
***Sleep disturbances***						
Emse 1	0.205	0.070	–0.346	–0.070	0.001	
Emse 2	0.217	0.046	0.128	0.307	0.001	
						0.152
Emco 1	0.215	0.098	–0.412	–0.025	0.013	
Emco 2	0.190	0.077	0.039	0.341	0.010	
						0.135
Seco 1	–0.010	0.101	–0.188	0.209	0.459	
Seco 2	–0.021	0.075	–0.169	0.127	0.800	
						0.015

*1 = Results from MI with Bayesian estimation, standardized by the mean of the latent variable in the first group, one tailed *p* values. 2 = Results from frequentist *t*-test comparison of index variables, two-tailed *p* values.*

## Discussion

Our aim with this study was twofold. First, we aimed to investigate whether scales commonly used to describe depressive symptoms, emotional exhaustion, and sleep can be validly used to compare mental health problems in three different groups of workers: employed workers, self-employed workers, and combinators. Second, we aimed to compare mean depressive symptoms, emotional exhaustion, and sleep disturbances in all three groups.

Based on approximate MI analyses, the scales assessing depressive symptoms and emotional exhaustion had acceptable fit on the scalar level. The scale assessing sleep disturbances had good fit on the scalar level. Thus, factor loadings and intercepts are invariant across groups and consequently comparable. In comparing the means of the three groups on the three constructs, we found minor to small differences between the three employment groups. The self-employed reported the lowest level of mental health problems with regard to depressive symptoms, mental exhaustion and sleep disturbances, and organizationally employed workers reported the highest levels of all three groups. Combinators were in between these two groups. The probability that the mean differences were not equal to zero was high for mean differences between employed and self-employed workers on all three constructs, as well as the mean difference in emotional exhaustion between self-employed workers and combinators, and the mean differences in sleep disturbances in employed workers and combinators.

### Mean Differences in Mental Health Problems in Workers

In terms of the mean comparisons, we found that self-employed workers reported less mental health problems than both employed workers and combinators. This is in line with Sikora and Saha (2009), as they found that self-employed workers experience less exhaustion, and with Rietveld et al. (2015), who found that self-employed workers experience less depressive symptoms. Further, this is also in line with other research, finding that self-employed workers are healthier than employed workers are (Bradley and Roberts, 2004; Stephan and Roesler, 2010; Pfeifer, 2013; Rietveld et al., 2015; Toivanen et al., 2016). We differentiated the group of combinators from both self-employed and organizationally employed workers, as they differ in traits and work characteristics from both of the other two groups. This group expressed less mental health problems than employed workers in terms of depressive symptoms, emotional exhaustion, and sleep disturbances, but more than self-employed workers. The findings imply that it is valuable to differentiate them as a group, who seem to benefit somewhat, but not as much as self-employed workers when it comes to mental health. It also means that the struggles to organize self-employment and organizational employment do not seem to be more tedious than just being organizationally employed.

Even though some mean differences in mental health seem to exist between groups, considering the size of the differences, none of them was substantial. Thus, the question needs to be raised whether these differences have practical meaning. From a theoretical perspective, this may indicate, contradictory to previous research, that despite the differences in who works in what employment type, and how work in these employments can be characterized, resources, demands (job-demand control model), and recovery (stressor-detachment theory) equal each other out for all three employment groups. However, the unsubstantially small differences between the groups of workers in mental health problems may lie in the variability within the groups, and that this variability is as large, or even larger, than that between groups. The variability between employed workers are large, and Bujacz et al. (2019) recently found that this is true for self-employed workers as well. If true, differences within the groups, for example, type of work, may cancel out any differences that researchers expect to find on the mean level.

What is important to note is that we here have studied symptoms of mental health problems, and that mental health problems can be assessed in many ways, for example, by looking at sick leave and doctors’ visits (Stephan and Roesler, 2010; Pfeifer, 2013). Rather than asking for differences in symptoms, as the current study did, these studies ask another question: “are self-employed workers less clinically ill than employed workers?”, and tend to find larger differences in mental health problems. These are not incompatible findings, as presence of symptoms and clinical mental health problems are not the same. Thus, unsubstantially small differences in mental health problem symptoms between groups of workers might not be an indication of unsubstantial small differences in clinical problems.

### Method of Comparison

In the BSEM CFAs, the scale assessing sleep disturbances fitted better than the other two. This might be due to several reasons: This scale is a behavioral scale, whereas the other two are affective. Behavioral scales generally have less structural problems, as the items are more often interpreted the same by respondents, but are often further away from the phenomena the researcher wants to assess. Further, the sleep disturbances scale is shorter than the other two. While it is difficult to build a short scale with good psychometric qualities, choosing few of the best indicators is plausible in structural equation modeling (Hayduk and Littvay, 2012). Lastly, to answer the items of the sleep disturbances scale, the respondents judge their average sleep over a longer period (3 months), whereas for the other two scales, the respondents relate to the last week or the present, introducing more variance. Hence, while the characteristics of the sleep disturbances scale have the advantage of a more general assessment of sleep during a longer period, the other two scales have the advantage of room for more variation and a momentary assessment of mental health problems. By using a mix of these types of scales, we provide a broad assessment of scales capturing mental health problems in different ways.

Using approximate MI tests enabled us to fit models that were closer to reality than traditional MI testing, which is in line with the argument by Van De Schoot et al. (2013): the assumption of zero differences is unrealistic. With traditional MI analyses, we would not have been able to compare the scales of depressive symptoms and emotional exhaustion between the three groups. Both scales had acceptable yet not very good fit when using the priors of BSEM. However, we were deliberately conservative when choosing priors, as we decided to use as small priors as possible instead of being satisfied with the larger ones yielded in the process suggested by Asparouhov and Muthén (2019). Our more conservative approach made sure not to allow any variance unrelated to the constructs. However, at the same time, it may be an explanation for the fact that fit was acceptable but not particularly good. Yet, even with this rather restrictive approach, both scales had acceptable fit on the scalar level and thus allowing for comparisons of means.

### Strengths, Limitations, and Contribution

There are many advantages to our study, and the strongest are as follows: we are using a national representative sample of workers, we compare three groups of workers with each other where others often overlook the group of combinators, and we are using rigid and careful statistical methods, using what modern statistics have to offer. Like in all studies, there are drawbacks too that have to be acknowledged. As many scales assessing mental health problems, our data are skewed, as the presence of depressive symptoms, emotional exhaustion, and sleep disturbances are not normally distributed in the population, but rather, most people are relatively healthy. Using Bayesian estimation precautions the problems using parametric analysis on skewed data, making the analysis more robust to skewness. We found this the most viable option, as non-parametric SEM is both difficult to interpret and not commonly used. Another limitation is that the group of combinators is rather small. We chose to run two types of comparisons when testing for approximate MI: one type including the combinators where we matched the sample sizes of employed and self-employed workers to the combinators (Emco and Seco models), and one type with larger samples without the combinators (Emse models). This way, we used the larger amount of data available for the employed and self-employed workers, adding power to the analyses.

The main contribution of this study is the use of Bayesian MI to test whether the three groups of workers actually can be compared on mental health measures. Based on evidence from Sweden, we provide an example of how researchers making these comparisons can conduct studies comparing self-employed workers, employed workers, and combinators being sure that results indicate actual group differences. Further, we contribute insight that when MI is controlled, differences between the three groups may in fact be substantially small and would likely disappear when controlled for demographic differences among the groups. Lastly, we distinguished the group of combinators because, given their differences from the other groups of workers, it is not justified to think of them as organizationally employed or self-employed.

We based this study on data collected in Sweden. In Sweden, self-employment is around 10% and combinators are rather common. The rate of self-employment thus is lower than in other countries, and this is believed to be explained by the fact that in Sweden, employment conditions are comparatively good so that self-employment is considered less as a viable option. Nevertheless, the group that chooses self-employment in Sweden may still be rather comparable with self-employed individuals in other countries, when background characteristics such as age, gender, region of birth, or education are considered. For example, the typical self-employed in Sweden is around 50 years old and without higher education, and this does not differ from the rest of Europe. Further, the constructs of depressive symptoms, emotional exhaustion, and sleep disturbances have been developed with the thought to be used internationally and are used widely in health studies across different nations and cultures. This means that the results of this study may be generalizable to other contexts to some extent, but a main message of this study is that MI should be tested across samples before any comparative analyses.

## Conclusion

In conclusion, we found that self-employed workers have slightly lower levels of depressive symptoms, emotional exhaustion, and sleep disturbances than combinators, and that the employed workers report highest levels of mental health problems of all three groups. However, these differences are substantially small and might have little practical meaning. This might be due to the large variability within groups. In future research, we would like to see that mental health problems of workers are studied with careful and thorough application of statistical methods, such as approximate MI analysis. Further, we think it is important that combinators continue to be studied separately. To test the idea that differences within groups are as large as differences between groups, more person-oriented approaches may be needed, exploring what subgroups of self-employed and combinators there may be with similar work and health profiles. Lastly, we would also like to see mental health of these groups of workers being studied from a broader perspective, to include positive mental health and affect while working, where differences may be found.

## Data Availability Statement

The raw data supporting the conclusions of this article will be made available by the authors upon resonable request.

## Ethics Statement

The studies involving human participants were reviewed and approved by Etikprövningsmyndigheten (the Ethics Review Authority Sweden). The patients/participants provided their written informed consent to participate in this study.

## Author Contributions

All authors collaborated in a research project that investigates self-employment and health. LB had the idea for the manuscript. All authors collaborated on discussing and structuring the idea and the manuscript. LB did all the analyses with assistance from AB. LB did the main part of the writing together with CB-O. All authors read, commented and added to the manuscript and approved its final version.

## Conflict of Interest

The authors declare that the research was conducted in the absence of any commercial or financial relationships that could be construed as a potential conflict of interest.
