# Supplementary material for: Comparing Depressive Symptoms, Emotional Exhaustion, and Sleep Disturbances in Self-Employed and Employed Workers: Application of Approximate Bayesian Measurement Invariance
Source: Front Psychol. 2021 Feb 2;11:598303. doi: 10.3389/fpsyg.2020.598303 (PMC7884631; doi:10.3389/fpsyg.2020.598303)
Supplement: Supplementary file 1 [file Data_Sheet_1.docx]

Supplementary Material

# Supplementary code

*Table 1. List of Mplus input files. In addition to the analyses listed below, we conducted all analyses with double amount of iterations and sensitivity analysis for the approximate measurement invariances analysis with different priors.*

| Input files | |
| --- | --- |
| 1 | Depressive symptoms, EM large: CFA |
| 2 | Depressive symptoms, EM large: CFA with priors |
| 3 | Depressive symptoms, SE large: CFA |
| 4 | Depressive symptoms, SE large: CFA with priors |
| 5 | Depressive symptoms, EM small: CFA |
| 6 | Depressive symptoms, EM small: CFA with priors |
| 7 | Depressive symptoms, SE small: CFA |
| 8 | Depressive symptoms, SE small: CFA with priors |
| 9 | Depressive symptoms, CO small: CFA |
| 10 | Depressive symptoms, CO small: CFA with priors |
| 11 | Emotional exhaustion, EM large: CFA |
| 12 | Emotional exhaustion, EM large: CFA with priors |
| 13 | Emotional exhaustion, SE large: CFA |
| 14 | Emotional exhaustion, SE large: CFA with priors |
| 15 | Emotional exhaustion, EM small: CFA |
| 16 | Emotional exhaustion, EM small: CFA with priors |
| 17 | Emotional exhaustion, SE small: CFA |
| 18 | Emotional exhaustion, SE small: CFA with priors |
| 19 | Emotional exhaustion, CO small: CFA |
| 20 | Emotional exhaustion, CO small: CFA with priors |
| 21 | Sleep disturbances, EM large: CFA |
| 22 | Sleep disturbances, SE large: CFA |
| 23 | Sleep disturbances, EM small: CFA |
| 24 | Sleep disturbances, SE small: CFA |
| 25 | Sleep disturbances, CO small: CFA |
| 26 | Depressive symptoms: EMSE Configural MI |
| 27 | Depressive symptoms: EMSE Metric MI |
| 28 | Depressive symptoms: EMSE Scalar MI |
| 29 | Depressive symptoms: EMCO Configural MI |
| 30 | Depressive symptoms: EMCO Metric MI |
| 31 | Depressive symptoms: EMCO Scalar MI |
| 32 | Depressive symptoms: SECO Configural MI |
| 33 | Depressive symptoms: SECO Metric MI |
| 34 | Depressive symptoms: SECO Scalar MI |
| 35 | Emotional exhaustion: EMSE Configural MI |
| 36 | Emotional exhaustion: EMSE Metric MI |
| 37 | Emotional exhaustion: EMSE Scalar MI |
| 38 | Emotional exhaustion: EMCO Configural MI |
| 39 | Emotional exhaustion: EMCO Metric MI |
| 40 | Emotional exhaustion: EMCO Scalar MI |
| 41 | Emotional exhaustion: SECO Configural MI |
| 42 | Emotional exhaustion: SECO Metric MI |
| 43 | Emotional exhaustion: SECO Scalar MI |
| 44 | Sleep disturbances: EMSE Configural MI |
| 45 | Sleep disturbances: EMSE Metric MI |
| 46 | Sleep disturbances: EMSE Scalar MI |
| 47 | Sleep disturbances: EMCO Configural MI |
| 48 | Sleep disturbances: EMCO Metric MI |
| 49 | Sleep disturbances: EMCO Scalar MI |
| 50 | Sleep disturbances: SECO Configural MI |
| 51 | Sleep disturbances: SECO Metric MI |
| 52 | Sleep disturbances: SECO Scalar MI |

# Supplementary tables

*Data preparation*

As samples of largely divergent sizes may bring unreliable results in comparison analyses, we drew five subsamples from the complete dataset. We matched these subsamples to the original data set with consideration to important background variables (gender, civil status, children, socioeconomic status, education level and region of birth). We let the smallest sample size of a comparison dictate subsample size. In comparisons between employed workers and self-employed workers, we used subsamples with 1034 participants in each group, and in comparisons between employed workers and combinators, and self-employed workers and combinators, we used subsamples 339 participants in each group.

*Table 1. Subsamples.*

|  | Salaried workers | Self-employed workers | Combinators |
| --- | --- | --- | --- |
| Sample size *n=1034* | EM large sample | SE large sample | - |
| Sample size *n=339* | EM small sample | SE small sample | CO small sample |

*EM =Employed workers. SE=Self-employed workers. CO=Combinators.*

*Table 2. Background information about each sample.*

|  | Full sample | | Large sample | | | Small sample | | |
| --- | --- | --- | --- | --- | --- | --- | --- | --- |
|  | All | EM | EM | SE | | EM | SE | CO |
|  | N=15634 | N=14254 | N= 1034 | | N= 1034 | N= 339 | N= 339 | N= 339 |
| *Background variables %* | | | | | | | | |
| Mean age (SD) | 51 (10) | 51 (10) | 51 (10) | 55 (10) | | 51 (10) | 55 (11) | 49 (11) |
| Female | 56.9 | 58.9 | 59.1 | 66.3 | | 58.8 | 67.8 | 55.5 |
| Male | 43.1 | 41.1 | 40.9 | 33.7 | | 41.2 | 32.2 | 44.5 |
| Married/cohabiting | 79.3 | 78.8 | 78.1 | 84.5 | | 76.0 | 83.8 | 80.5 |
| Single | 20.7 | 21.2 | 21.9 | 15.5 | | 24.0 | 16.2 | 19.5 |
| Living with children | 44.9 | 46.0 | 46.7 | 37.9 | | 42.9 | 39.3 | 49.7 |
| **Highest education** |  |  |  |  | |  |  |  |
| Elementary school | 10.3 | 10.2 | 10.4 | 12.5 | | 9.6 | 12.8 | 7.5 |
| 2-year HS*/training school | 21.3 | 21.1 | 21 | 24.1 | | 20.9 | 21.2 | 17.6 |
| 3 - or 4-year HS* | 23.6 | 23.0 | 20.6 | 31.2 | | 22.4 | 31.6 | 26.3 |
| University < 3 years | 15 | 15.4 | 16.8 | 9.5 | | 16.7 | 9.6 | 14.6 |
| University ≥ 3 years | 29.8 | 30.2 | 31.3 | 22.7 | | 30.4 | 24.8 | 34 |
| **Country of birth** |  |  |  |  | |  |  |  |
| Sweden | 93.5 | 93.3 | 93.5 | 95.7 | | 94.3 | 95.8 | 95.8 |
| Other Nordic countries | 2.7 | 2.8 | 2.8 | 1.8 | | 2.7 | 0.9 | 2.1 |
| Other European countries | 2.3 | 2.4 | 2.5 | 1.6 | | 2.4 | 2.4 | 1.5 |
| Africa | 0.2 | 0.2 | 0.4 | 0 | | 0 | 0 | 0 |
| Asia | 0.9 | 0.9 | 0.5 | 0.7 | | 0.3 | 0.6 | 0.6 |
| North America | 0.1 | 0.1 | 0.2 | 0.3 | | 0 | 0.3 | 0 |
| South America | 0.3 | 0.3 | 0.2 | 0 | | 0.3 | 0 | 0 |

**High school*

*Scalar model loadings and cross loadings*

*Table 3. Depressive symptoms scalar model loadings and cross loadings.*

|  |  | | | |  | |  | | | 95% CI | |
| --- | --- | --- | --- | --- | --- | --- | --- | --- | --- | --- | --- |
|  | | Estimate | | Posterior SD | | One-tailed p-value | | Lower 2.5% | | | Upper 2.5% |
| *Employed and self-employed workers* | | | | | | | | | | | |
| Employed workers | | |  | | |  | | |  | | |
| *Depressive symptoms by* | | | |  | | | |  | | | |
| SCL-CD_6_1 | | 0.764 | | 0.031 | | 0.000 | | 0.704 | | | 0.825 |
| SCL-CD_6_2 | | 0.730 | | 0.025 | | 0.000 | | 0.682 | | | 0.781 |
| SCL-CD_6_3 | | 0.691 | | 0.029 | | 0.000 | | 0.636 | | | 0.747 |
| SCL-CD_6_4 | | 0.851 | | 0.032 | | 0.000 | | 0.790 | | | 0.914 |
| SCL-CD_6_5 | | 0.739 | | 0.027 | | 0.000 | | 0.686 | | | 0.794 |
| SCL-CD_6_6 | | 0.871 | | 0.028 | | 0.000 | | 0.816 | | | 0.928 |
| *SCL-CD6_1_ with* | | |  | | |  | | |  | | |
| SCL-CD_6_2 | | 0.009 | | 0.013 | | 0.252 | | -0.016 | | | 0.034 |
| SCL-CD_6_3 | | -0.062 | | 0.014 | | 0.000 | | -0.090 | | | -0.034 |
| SCL-CD_6_4 | | -0.026 | | 0.016 | | 0.051 | | -0.057 | | | 0.005 |
| SCL-CD_6_5 | | 0.008 | | 0.014 | | 0.280 | | -0.019 | | | 0.037 |
| SCL-CD_6_6 | | 0.043 | | 0.015 | | 0.001 | | 0.015 | | | 0.074 |
| *SCL-CD_6_2 with* | | |  | | |  | | |  | | |
| SCL-CD_6_3 | | 0.013 | | 0.012 | | 0.139 | | -0.010 | | | 0.037 |
| SCL-CD_6_4 | | 0.002 | | 0.013 | | 0.452 | | -0.023 | | | 0.027 |
| SCL-CD_6_5 | | 0.008 | | 0.011 | | 0.228 | | -0.013 | | | 0.030 |
| SCL-CD_6_6 | | -0.018 | | 0.010 | | 0.040 | | -0.038 | | | 0.002 |
| *SCL-CD_6_3 with* |  | | | |  | |  | | |  | |
| SCL-CD_6_4 | | 0.094 | | 0.017 | | 0.000 | | 0.063 | | | 0.128 |
| SCL-CD_6_5 | | -0.015 | | 0.013 | | 0.114 | | -0.040 | | | 0.010 |
| SCL-CD_6_6 | | -0.026 | | 0.013 | | 0.021 | | -0.050 | | | -0.001 |
| *SCL-CD_6_4 with* | | |  | | |  | | |  | | |
| SCL-CD_6_5 | | -0.038 | | 0.013 | | 0.002 | | -0.064 | | | -0.012 |
| SCL-CD_6_6 | | -0.011 | | 0.014 | | 0.218 | | -0.037 | | | 0.017 |
| *SCL-CD_6_5 with* |  | | | |  | |  | | |  | |
| SCL-CD_6_6 | | 0.025 | | 0.013 | | 0.018 | | 0.002 | | | 0.051 |
| Self-employed workers | | |  | | |  | | |  | | |
| *Depressive symptoms by* | | | |  | | | |  | | | |
| SCL-CD_6_1 | | 0.778 | | 0.050 | | 0.000 | | 0.683 | | | 0.878 |
| SCL-CD_6_2 | | 0.783 | | 0.048 | | 0.000 | | 0.689 | | | 0.879 |
| SCL-CD_6_3 | | 0.675 | | 0.045 | | 0.000 | | 0.590 | | | 0.765 |
| SCL-CD_6_4 | | 0.838 | | 0.053 | | 0.000 | | 0.736 | | | 0.944 |
| SCL-CD_6_5 | | 0.738 | | 0.047 | | 0.000 | | 0.648 | | | 0.831 |
| SCL-CD_6_6 | | 0.857 | | 0.053 | | 0.000 | | 0.754 | | | 0.961 |
| SCL-CD_6_1  *with* | | |  | | |  | | |  | | |
| SCL-CD_6_2 | | 0.024 | | 0.012 | | 0.021 | | 0.001 | | | 0.048 |
| SCL-CD_6_3 | | -0.030 | | 0.014 | | 0.017 | | -0.056 | | | -0.002 |
| SCL-CD_6_4 | | -0.039 | | 0.014 | | 0.004 | | -0.067 | | | -0.011 |
| SCL-CD_6_5 | | 0.009 | | 0.012 | | 0.231 | | -0.015 | | | 0.034 |
| SCL-CD_6_6 | | 0.010 | | 0.012 | | 0.202 | | -0.013 | | | 0.035 |
| SCL-CD_6_2  *with* | | |  | | |  | | |  | | |
| SCL-CD_6_3 | | 0.022 | | 0.012 | | 0.029 | | -0.001 | | | 0.047 |
| SCL-CD_6_4 | | 0.002 | | 0.012 | | 0.445 | | -0.022 | | | 0.026 |
| SCL-CD_6_5 | | -0.002 | | 0.010 | | 0.410 | | -0.021 | | | 0.018 |
| SCL-CD_6_6 | | -0.021 | | 0.009 | | 0.014 | | -0.038 | | | -0.002 |
| *SCL-CD_6_3 with* |  | | | |  | |  | | |  | |
| SCL-CD_6_4 | | 0.086 | | 0.016 | | 0.000 | | 0.056 | | | 0.119 |
| SCL-CD_6_5 | | -0.032 | | 0.012 | | 0.004 | | -0.054 | | | -0.009 |
| SCL-CD_6_6 | | -0.027 | | 0.012 | | 0.012 | | -0.049 | | | -0.004 |
| *SCL-CD_6_4 with* | | |  | | |  | | |  | | |
| SCL-CD_6_5 | | -0.032 | | 0.012 | | 0.005 | | -0.055 | | | -0.008 |
| SCL-CD_6_6 | | 0.006 | | 0.012 | | 0.315 | | -0.018 | | | 0.031 |
| *SCL-CD_6_5 with* |  | | | |  | |  | | |  | |
| SCL-CD_6_6 | | 0.032 | | 0.011 | | 0.001 | | 0.012 | | | 0.054 |
| Employed workers and combinators | | | | | | | | | | | |
|  |  | | | |  | |  | | | 95% CI | |
|  | | Estimate | | Posterior SD | | One-tailed p-value | | Lower 2.5% | | | Upper 2.5% |
| Employed workers | | |  | | |  | | |  | | |
| *Depressive symptoms by* | | | |  | | | |  | | | |
| SCL-CD_6_1 | | 0.817 | | 0.051 | | 0.000 | | 0.722 | | | 0.922 |
| SCL-CD_6_2 | | 0.865 | | 0.047 | | 0.000 | | 0.778 | | | 0.961 |
| SCL-CD_6_3 | | 0.716 | | 0.049 | | 0.000 | | 0.624 | | | 0.817 |
| SCL-CD_6_4 | | 0.952 | | 0.055 | | 0.000 | | 0.849 | | | 1.064 |
| SCL-CD_6_5 | | 0.817 | | 0.046 | | 0.000 | | 0.730 | | | 0.912 |
| SCL-CD_6_6 | | 0.972 | | 0.047 | | 0.000 | | 0.884 | | | 1.070 |
| *SCL-CD6_1_ with* | | |  | | |  | | |  | | |
| SCL-CD_6_2 | | 0.003 | | 0.009 | | 0.361 | | -0.014 | | | 0.021 |
| SCL-CD_6_3 | | -0.007 | | 0.011 | | 0.266 | | -0.029 | | | 0.015 |
| SCL-CD_6_4 | | -0.004 | | 0.011 | | 0.368 | | -0.026 | | | 0.018 |
| SCL-CD_6_5 | | 0.002 | | 0.009 | | 0.428 | | -0.017 | | | 0.020 |
| SCL-CD_6_6 | | 0.001 | | 0.008 | | 0.441 | | -0.014 | | | 0.017 |
| *SCL-CD_6_2 with* | | |  | | |  | | |  | | |
| SCL-CD_6_3 | | 0.001 | | 0.009 | | 0.439 | | -0.017 | | | 0.020 |
| SCL-CD_6_4 | | 0.000 | | 0.009 | | 0.493 | | -0.018 | | | 0.018 |
| SCL-CD_6_5 | | 0.001 | | 0.008 | | 0.450 | | -0.014 | | | 0.016 |
| SCL-CD_6_6 | | 0.002 | | 0.006 | | 0.383 | | -0.014 | | | 0.011 |
| *SCL-CD_6_3 with* |  | | | |  | |  | | |  | |
| SCL-CD_6_4 | | 0.020 | | 0.012 | | 0.044 | | -0.003 | | | 0.043 |
| SCL-CD_6_5 | | -0.004 | | 0.009 | | 0.318 | | -0.023 | | | 0.014 |
| SCL-CD_6_6 | | -0.004 | | 0.008 | | 0.293 | | -0.020 | | | 0.011 |
| *SCL-CD_6_4 with* | | |  | | |  | | |  | | |
| SCL-CD_6_5 | | -0.008 | | 0.009 | | 0.184 | | -0.027 | | | 0.010 |
| SCL-CD_6_6 | | 0.000 | | 0.008 | | 0.493 | | -0.015 | | | 0.016 |
| *SCL-CD_6_5 with* |  | | | |  | |  | | |  | |
| SCL-CD_6_6 | | 0.004 | | 0.007 | | 0.249 | | -0.008 | | | 0.017 |
| Combinators |  | | | |  | |  | | |  | |
| *Depressive symptoms by* | | | |  | | | |  | | | |
| SCL-CD_6_1 | | 0.868 | | 0.069 | | 0.000 | | 0.737 | | | 1.010 |
| SCL-CD_6_2 | | 0.820 | | 0.063 | | 0.000 | | 0.700 | | | 0.948 |
| SCL-CD_6_3 | | 0.768 | | 0.061 | | 0.000 | | 0.654 | | | 0.893 |
| SCL-CD_6_4 | | 0.943 | | 0.072 | | 0.000 | | 0.807 | | | 1.088 |
| SCL-CD_6_5 | | 0.822 | | 0.065 | | 0.000 | | 0.700 | | | 0.954 |
| SCL-CD_6_6 | | 0.941 | | 0.070 | | 0.000 | | 0.809 | | | 1.083 |
| SCL-CD_6_1  *with* | | |  | | |  | | |  | | |
| SCL-CD_6_2 | | 0.009 | | 0.015 | | 0.264 | | -0.020 | | | 0.039 |
| SCL-CD_6_3 | | -0.023 | | 0.015 | | 0.067 | | -0.053 | | | 0.007 |
| SCL-CD_6_4 | | -0.015 | | 0.017 | | 0.203 | | -0.049 | | | 0.020 |
| SCL-CD_6_5 | | -0.006 | | 0.017 | | 0.367 | | -0.038 | | | 0.027 |
| SCL-CD_6_6 | | 0.030 | | 0.016 | | 0.030 | | -0.001 | | | 0.063 |
| SCL-CD_6_2  *with* | | |  | | |  | | |  | | |
| SCL-CD_6_3 | | 0.004 | | 0.012 | | 0.364 | | -0.019 | | | 0.027 |
| SCL-CD_6_4 | | -0.001 | | 0.013 | | 0.475 | | -0.027 | | | 0.025 |
| SCL-CD_6_5 | | 0.009 | | 0.013 | | 0.249 | | -0.016 | | | 0.033 |
| SCL-CD_6_6 | | -0.014 | | 0.012 | | 0.117 | | -0.038 | | | 0.009 |
| *SCL-CD_6_3 with* |  | | | |  | |  | | |  | |
| SCL-CD_6_4 | | 0.022 | | 0.014 | | 0.061 | | -0.006 | | | 0.050 |
| SCL-CD_6_5 | | 0.005 | | 0.013 | | 0.347 | | -0.020 | | | 0.031 |
| SCL-CD_6_6 | | -0.010 | | 0.013 | | 0.221 | | -0.034 | | | 0.015 |
| *SCL-CD_6_4 with* | | |  | | |  | | |  | | |
| SCL-CD_6_5 | | -0.013 | | 0.015 | | 0.180 | | -0.042 | | | 0.015 |
| SCL-CD_6_6 | | 0.004 | | 0.014 | | 0.391 | | -0.024 | | | 0.032 |
| *SCL-CD_6_5 with* |  | | | |  | |  | | |  | |
| SCL-CD_6_6 | | 0.004 | | 0.014 | | 0.396 | | -0.023 | | | 0.030 |
| Self-employed workers and combinators | | | | | | | | | | | |
|  |  | | | |  | |  | | | 95% CI | |
|  | | Estimate | | Posterior SD | | One-tailed p-value | | Lower 2.5% | | | Upper 2.5% |
| Self-employed workers | | |  | | |  | | |  | | |
| *Depressive symptoms by* | | | |  | | | |  | | | |
| SCL-CD_6_1 | | 0.807 | | 0.053 | | 0.000 | | 0.707 | | | 0.916 |
| SCL-CD_6_2 | | 0.820 | | 0.048 | | 0.000 | | 0.729 | | | 0.917 |
| SCL-CD_6_3 | | 0.716 | | 0.050 | | 0.000 | | 0.621 | | | 0.818 |
| SCL-CD_6_4 | | 0.842 | | 0.056 | | 0.000 | | 0.736 | | | 0.955 |
| SCL-CD_6_5 | | 0.794 | | 0.044 | | 0.000 | | 0.713 | | | 0.884 |
| SCL-CD_6_6 | | 0.886 | | 0.051 | | 0.000 | | 0.790 | | | 0.991 |
| *SCL-CD6_1_ with* | | |  | | |  | | |  | | |
| SCL-CD_6_2 | | 0.007 | | 0.018 | | 0.355 | | -0.028 | | | 0.043 |
| SCL-CD_6_3 | | -0.021 | | 0.020 | | 0.155 | | -0.060 | | | 0.020 |
| SCL-CD_6_4 | | -0.029 | | 0.022 | | 0.094 | | -0.072 | | | 0.015 |
| SCL-CD_6_5 | | 0.010 | | 0.016 | | 0.267 | | -0.021 | | | 0.042 |
| SCL-CD_6_6 | | 0.017 | | 0.020 | | 0.187 | | -0.020 | | | 0.057 |
| *SCL-CD_6_2 with* | | |  | | |  | | |  | | |
| SCL-CD_6_3 | | 0.045 | | 0.018 | | 0.005 | | 0.010 | | | 0.082 |
| SCL-CD_6_4 | | 0.002 | | 0.019 | | 0.450 | | -0.034 | | | 0.041 |
| SCL-CD_6_5 | | 0.000 | | 0.013 | | 0.490 | | -0.026 | | | 0.027 |
| SCL-CD_6_6 | | -0.032 | | 0.016 | | 0.021 | | -0.063 | | | -0.001 |
| *SCL-CD_6_3 with* |  | | | |  | |  | | |  | |
| SCL-CD_6_4 | | 0.061 | | 0.023 | | 0.003 | | 0.018 | | | 0.106 |
| SCL-CD_6_5 | | -0.034 | | 0.015 | | 0.013 | | -0.063 | | | -0.004 |
| SCL-CD_6_6 | | -0.024 | | 0.018 | | 0.096 | | -0.059 | | | 0.012 |
| *SCL-CD_6_4 with* | | |  | | |  | | |  | | |
| SCL-CD_6_5 | | -0.015 | | 0.016 | | 0.174 | | -0.047 | | | 0.017 |
| SCL-CD_6_6 | | 0.002 | | 0.020 | | 0.468 | | -0.037 | | | 0.042 |
| *SCL-CD_6_5 with* |  | | | |  | |  | | |  | |
| SCL-CD_6_6 | | 0.030 | | 0.015 | | 0.018 | | 0.002 | | | 0.061 |
| Combinators |  | | | |  | |  | | |  | |
| *Depressive symptoms by* | | | |  | | | |  | | | |
| SCL-CD_6_1 | | 0.864 | | 0.141 | | 0.000 | | 0.597 | | | 1.150 |
| SCL-CD_6_2 | | 0.798 | | 0.129 | | 0.000 | | 0.554 | | | 1.058 |
| SCL-CD_6_3 | | 0.762 | | 0.124 | | 0.000 | | 0.527 | | | 1.012 |
| SCL-CD_6_4 | | 0.922 | | 0.148 | | 0.000 | | 0.640 | | | 1.218 |
| SCL-CD_6_5 | | 0.807 | | 0.131 | | 0.000 | | 0.559 | | | 1.072 |
| SCL-CD_6_6 | | 0.920 | | 0.147 | | 0.000 | | 0.639 | | | 1.215 |
| SCL-CD_6_1  *with* | | |  | | |  | | |  | | |
| SCL-CD_6_2 | | 0.009 | | 0.015 | | 0.267 | | -0.020 | | | 0.039 |
| SCL-CD_6_3 | | -0.023 | | 0.015 | | 0.063 | | -0.054 | | | 0.007 |
| SCL-CD_6_4 | | -0.015 | | 0.017 | | 0.199 | | -0.049 | | | 0.020 |
| SCL-CD_6_5 | | -0.006 | | 0.017 | | 0.361 | | -0.038 | | | 0.027 |
| SCL-CD_6_6 | | 0.030 | | 0.016 | | 0.030 | | -0.001 | | | 0.062 |
| SCL-CD_6_2  *with* | | |  | | |  | | |  | | |
| SCL-CD_6_3 | | 0.004 | | 0.012 | | 0.364 | | -0.019 | | | 0.027 |
| SCL-CD_6_4 | | -0.001 | | 0.013 | | 0.482 | | -0.026 | | | 0.026 |
| SCL-CD_6_5 | | 0.009 | | 0.013 | | 0.246 | | -0.016 | | | 0.033 |
| SCL-CD_6_6 | | -0.014 | | 0.012 | | 0.121 | | -0.037 | | | 0.009 |
| *SCL-CD_6_3 with* |  | | | |  | |  | | |  | |
| SCL-CD_6_4 | | 0.022 | | 0.014 | | 0.062 | | -0.006 | | | 0.049 |
| SCL-CD_6_5 | | 0.005 | | 0.013 | | 0.351 | | -0.021 | | | 0.031 |
| SCL-CD_6_6 | | -0.010 | | 0.013 | | 0.219 | | -0.034 | | | 0.015 |
| *SCL-CD_6_4 with* | | |  | | |  | | |  | | |
| SCL-CD_6_5 | | -0.013 | | 0.015 | | 0.182 | | -0.042 | | | 0.016 |
| SCL-CD_6_6 | | 0.004 | | 0.014 | | 0.386 | | -0.024 | | | 0.033 |
| *SCL-CD_6_5 with* |  | | | |  | |  | | |  | |
| SCL-CD_6_6 | | 0.004 | | 0.014 | | 0.393 | | -0.023 | | | 0.030 |

*Table 4. Emotional exhaustion scalar model loadings and cross loadings.*

|  |  | | | |  | |  | | | 95% CI | |
| --- | --- | --- | --- | --- | --- | --- | --- | --- | --- | --- | --- |
|  | | Estimate | | Posterior SD | | One-tailed p-value | | Lower 2.5% | | | Upper 2.5% |
| Employed and self-employed workers | | | | | | | | | | | |
| Employed workers | | |  | | |  | | |  | | |
| *Emotional exhaustion by* | | |  | | |  | | |  | | |
| SMBQ1 | | 1.120 | | 0.044 | | 0.000 | | 1.036 | | | 1.207 |
| SMBQ2 | | 1.246 | | 0.041 | | 0.000 | | 1.168 | | | 1.329 |
| SMBQ3 | | 1.497 | | 0.039 | | 0.000 | | 1.424 | | | 1.576 |
| SMBQ4 | | 1.194 | | 0.037 | | 0.000 | | 1.122 | | | 1.269 |
| SMBQ5 | | 1.428 | | 0.041 | | 0.000 | | 1.349 | | | 1.510 |
| SMBQ6 | | 0.917 | | 0.044 | | 0.000 | | 0.832 | | | 1.005 |
| *SMBQ1 with* |  | | | |  | |  | | |  | |
| SMBQ2 | | -0.065 | | 0.028 | | 0.009 | | -0.119 | | | -0.011 |
| SMBQ3 | | 0.016 | | 0.021 | | 0.215 | | -0.024 | | | 0.057 |
| SMBQ4 | | -0.051 | | 0.023 | | 0.015 | | -0.097 | | | -0.005 |
| SMBQ5 | | 0.011 | | 0.025 | | 0.334 | | -0.037 | | | 0.060 |
| SMBQ6 | | 0.196 | | 0.037 | | 0.000 | | 0.126 | | | 0.269 |
| *SMBQ2 with* |  | | | |  | |  | | |  | |
| SMBQ3 | | 0.025 | | 0.017 | | 0.074 | | -0.009 | | | 0.060 |
| SMBQ4 | | 0.007 | | 0.020 | | 0.367 | | -0.032 | | | 0.048 |
| SMBQ5 | | -0.017 | | 0.021 | | 0.211 | | -0.057 | | | 0.024 |
| SMBQ6 | | -0.015 | | 0.030 | | 0.309 | | -0.074 | | | 0.045 |
| *SMBQ3 with* |  | | | |  | |  | | |  | |
| SMBQ4 | | 0.001 | | 0.014 | | 0.469 | | -0.026 | | | 0.030 |
| SMBQ5 | | -0.006 | | 0.014 | | 0.344 | | -0.033 | | | 0.023 |
| SMBQ6 | | -0.052 | | 0.022 | | 0.010 | | -0.095 | | | -0.008 |
| *SMBQ4 with* |  | | | |  | |  | | |  | |
| SMBQ5 | | 0.013 | | 0.018 | | 0.236 | | -0.022 | | | 0.049 |
| SMBQ6 | | 0.003 | | 0.026 | | 0.459 | | -0.048 | | | 0.054 |
| *SMBQ5 with* |  | | | |  | |  | | |  | |
| SMBQ6 | | 0.031 | | 0.027 | | 0.126 | | -0.022 | | | 0.085 |
| Self-employed workers | | |  | | |  | | |  | | |
| *Emotional exhaustion by* | | |  | | |  | | |  | | |
| SMBQ1 | | 1.051 | | 0.055 | | 0.000 | | 0.946 | | | 1.162 |
| SMBQ2 | | 1.194 | | 0.057 | | 0.000 | | 1.085 | | | 1.310 |
| SMBQ3 | | 1.474 | | 0.064 | | 0.000 | | 1.351 | | | 1.601 |
| SMBQ4 | | 1.300 | | 0.057 | | 0.000 | | 1.190 | | | 1.415 |
| SMBQ5 | | 1.474 | | 0.064 | | 0.000 | | 1.351 | | | 1.602 |
| SMBQ6 | | 0.896 | | 0.049 | | 0.000 | | 0.802 | | | 0.995 |
| *SMBQ1 with* |  | | | |  | |  | | |  | |
| SMBQ2 | | -0.054 | | 0.030 | | 0.036 | | -0.113 | | | 0.005 |
| SMBQ3 | | 0.028 | | 0.023 | | 0.101 | | -0.015 | | | 0.073 |
| SMBQ4 | | -0.091 | | 0.023 | | 0.000 | | -0.136 | | | -0.046 |
| SMBQ5 | | 0.024 | | 0.025 | | 0.162 | | -0.024 | | | 0.073 |
| SMBQ6 | | 0.136 | | 0.032 | | 0.000 | | 0.074 | | | 0.201 |
| *SMBQ2 with* |  | | | |  | |  | | |  | |
| SMBQ3 | | 0.031 | | 0.020 | | 0.055 | | -0.007 | | | 0.071 |
| SMBQ4 | | 0.010 | | 0.021 | | 0.314 | | -0.030 | | | 0.052 |
| SMBQ5 | | -0.034 | | 0.021 | | 0.055 | | -0.075 | | | 0.008 |
| SMBQ6 | | 0.004 | | 0.028 | | 0.438 | | -0.050 | | | 0.060 |
| *SMBQ3 with* |  | | | |  | |  | | |  | |
| SMBQ4 | | -0.002 | | 0.014 | | 0.453 | | -0.029 | | | 0.027 |
| SMBQ5 | | -0.011 | | 0.015 | | 0.232 | | -0.039 | | | 0.018 |
| SMBQ6 | | -0.025 | | 0.020 | | 0.109 | | -0.065 | | | 0.015 |
| *SMBQ4 with* |  | | | |  | |  | | |  | |
| SMBQ5 | | 0.031 | | 0.017 | | 0.029 | | -0.001 | | | 0.065 |
| SMBQ6 | | -0.003 | | 0.022 | | 0.446 | | -0.045 | | | 0.040 |
| *SMBQ5 with* |  | | | |  | |  | | |  | |
| SMBQ6 | | -0.009 | | 0.023 | | 0.351 | | -0.053 | | | 0.036 |
| Employed workers and combinators | | | | | | | | | | | |
|  |  | | | |  | |  | | | 95% CI | |
|  | | Estimate | | Posterior SD | | One-tailed p-value | | Lower 2.5% | | | Upper 2.5% |
| Employed workers | | |  | | |  | | |  | | |
| *Emotional exhaustion by* | | |  | | |  | | |  | | |
| SMBQ1 | | 0.982 | | 0.072 | | 0.000 | | 0.846 | | | 1.128 |
| SMBQ2 | | 1.151 | | 0.070 | | 0.000 | | 1.020 | | | 1.294 |
| SMBQ3 | | 1.431 | | 0.066 | | 0.000 | | 1.308 | | | 1.567 |
| SMBQ4 | | 1.205 | | 0.062 | | 0.000 | | 1.089 | | | 1.333 |
| SMBQ5 | | 1.380 | | 0.069 | | 0.000 | | 1.252 | | | 1.522 |
| SMBQ6 | | 0.889 | | 0.074 | | 0.000 | | 0.749 | | | 1.039 |
| *SMBQ1 with* |  | | | |  | |  | | |  | |
| SMBQ2 | | -0.007 | | 0.027 | | 0.398 | | -0.061 | | | 0.047 |
| SMBQ3 | | -0.001 | | 0.018 | | 0.479 | | -0.036 | | | 0.034 |
| SMBQ4 | | -0.014 | | 0.021 | | 0.254 | | -0.056 | | | 0.027 |
| SMBQ5 | | 0.004 | | 0.022 | | 0.432 | | -0.040 | | | 0.047 |
| SMBQ6 | | 0.065 | | 0.036 | | 0.032 | | -0.004 | | | 0.135 |
| *SMBQ2 with* |  | | | |  | |  | | |  | |
| SMBQ3 | | 0.005 | | 0.015 | | 0.381 | | -0.025 | | | 0.034 |
| SMBQ4 | | 0.013 | | 0.018 | | 0.230 | | -0.022 | | | 0.049 |
| SMBQ5 | | -0.018 | | 0.019 | | 0.167 | | -0.054 | | | 0.019 |
| SMBQ6 | | -0.002 | | 0.030 | | 0.478 | | -0.060 | | | 0.057 |
| *SMBQ3 with* |  | | | |  | |  | | |  | |
| SMBQ4 | | -0.002 | | 0.011 | | 0.418 | | -0.025 | | | 0.020 |
| SMBQ5 | | 0.004 | | 0.012 | | 0.360 | | -0.019 | | | 0.028 |
| SMBQ6 | | -0.012 | | 0.019 | | 0.272 | | -0.050 | | | 0.026 |
| *SMBQ4 with* |  | | | |  | |  | | |  | |
| SMBQ5 | | 0.003 | | 0.014 | | 0.416 | | -0.025 | | | 0.031 |
| SMBQ6 | | 0.001 | | 0.023 | | 0.481 | | -0.044 | | | 0.047 |
| *SMBQ5 with* |  | | | |  | |  | | |  | |
| SMBQ6 | | -0.001 | | 0.024 | | 0.483 | | -0.048 | | | 0.046 |
| Combinators |  | | | |  | |  | | |  | |
| *Emotional exhaustion by* | | |  | | |  | | |  | | |
| SMBQ1 | | 0.979 | | 0.079 | | 0.000 | | 0.831 | | | 1.139 |
| SMBQ2 | | 1.225 | | 0.080 | | 0.000 | | 1.073 | | | 1.387 |
| SMBQ3 | | 1.462 | | 0.087 | | 0.000 | | 1.298 | | | 1.637 |
| SMBQ4 | | 1.131 | | 0.074 | | 0.000 | | 0.991 | | | 1.283 |
| SMBQ5 | | 1.353 | | 0.084 | | 0.000 | | 1.194 | | | 1.524 |
| SMBQ6 | | 0.892 | | 0.076 | | 0.000 | | 0.748 | | | 1.047 |
| *SMBQ1 with* |  | | | |  | |  | | |  | |
| SMBQ2 | | -0.058 | | 0.037 | | 0.057 | | -0.131 | | | 0.014 |
| SMBQ3 | | 0.009 | | 0.028 | | 0.373 | | -0.045 | | | 0.064 |
| SMBQ4 | | -0.009 | | 0.031 | | 0.386 | | -0.071 | | | 0.053 |
| SMBQ5 | | 0.007 | | 0.032 | | 0.411 | | -0.056 | | | 0.071 |
| SMBQ6 | | 0.094 | | 0.047 | | 0.023 | | 0.002 | | | 0.188 |
| *SMBQ2 with* |  | | | |  | |  | | |  | |
| SMBQ3 | | 0.029 | | 0.022 | | 0.091 | | -0.014 | | | 0.074 |
| SMBQ4 | | -0.005 | | 0.025 | | 0.415 | | -0.054 | | | 0.044 |
| SMBQ5 | | 0.000 | | 0.025 | | 0.499 | | -0.050 | | | 0.050 |
| SMBQ6 | | -0.050 | | 0.037 | | 0.087 | | -0.123 | | | 0.022 |
| *SMBQ3 with* |  | | | |  | |  | | |  | |
| SMBQ4 | | -0.011 | | 0.018 | | 0.280 | | -0.046 | | | 0.025 |
| SMBQ5 | | -0.005 | | 0.019 | | 0.388 | | -0.042 | | | 0.032 |
| SMBQ6 | | -0.017 | | 0.028 | | 0.268 | | -0.071 | | | 0.038 |
| *SMBQ4 with* |  | | | |  | |  | | |  | |
| SMBQ5 | | 0.012 | | 0.022 | | 0.294 | | -0.030 | | | 0.055 |
| SMBQ6 | | 0.041 | | 0.032 | | 0.094 | | -0.020 | | | 0.104 |
| *SMBQ5 with* |  | | | |  | |  | | |  | |
| SMBQ6 | | -0.005 | | 0.032 | | 0.444 | | -0.068 | | | 0.059 |
| Self-employed workers and combinators | | | | | | | | | | | |
|  |  | | | |  | |  | | | 95% CI | |
|  | | Estimate | | Posterior SD | | One-tailed p-value | | Lower 2.5% | | | Upper 2.5% |
| Employed workers | | |  | | |  | | |  | | |
| *Emotional exhaustion by* | | |  | | |  | | |  | | |
| SMBQ1 | | 1.018 | | 0.070 | | 0.000 | | 0.885 | | | 1.161 |
| SMBQ2 | | 1.097 | | 0.068 | | 0.000 | | 0.969 | | | 1.237 |
| SMBQ3 | | 1.436 | | 0.065 | | 0.000 | | 1.316 | | | 1.572 |
| SMBQ4 | | 1.196 | | 0.060 | | 0.000 | | 1.085 | | | 1.320 |
| SMBQ5 | | 1.374 | | 0.067 | | 0.000 | | 1.249 | | | 1.513 |
| SMBQ6 | | 0.844 | | 0.068 | | 0.000 | | 0.715 | | | 0.981 |
| *SMBQ1 with* |  | | | |  | |  | | |  | |
| SMBQ2 | | -0.005 | | 0.020 | | 0.391 | | -0.044 | | | 0.033 |
| SMBQ3 | | 0.002 | | 0.012 | | 0.429 | | -0.022 | | | 0.027 |
| SMBQ4 | | -0.008 | | 0.014 | | 0.281 | | -0.037 | | | 0.020 |
| SMBQ5 | | 0.001 | | 0.015 | | 0.479 | | -0.029 | | | 0.031 |
| SMBQ6 | | 0.028 | | 0.023 | | 0.110 | | -0.017 | | | 0.074 |
| *SMBQ2 with* |  | | | |  | |  | | |  | |
| SMBQ3 | | 0.003 | | 0.011 | | 0.394 | | -0.018 | | | 0.024 |
| SMBQ4 | | 0.002 | | 0.013 | | 0.427 | | -0.022 | | | 0.027 |
| SMBQ5 | | -0.007 | | 0.013 | | 0.296 | | -0.033 | | | 0.019 |
| SMBQ6 | | 0.004 | | 0.020 | | 0.413 | | -0.035 | | | 0.044 |
| *SMBQ3 with* |  | | | |  | |  | | |  | |
| SMBQ4 | | 0.000 | | 0.008 | | 0.495 | | -0.015 | | | 0.015 |
| SMBQ5 | | 0.001 | | 0.008 | | 0.452 | | -0.015 | | | 0.017 |
| SMBQ6 | | -0.009 | | 0.013 | | 0.233 | | -0.034 | | | 0.015 |
| *SMBQ4 with* |  | | | |  | |  | | |  | |
| SMBQ5 | | 0.002 | | 0.010 | | 0.417 | | -0.017 | | | 0.021 |
| SMBQ6 | | 0.003 | | 0.015 | | 0.411 | | -0.025 | | | 0.032 |
| *SMBQ5 with* |  | | | |  | |  | | |  | |
| SMBQ6 | | 0.000 | | 0.016 | | 0.494 | | -0.030 | | | 0.031 |
| Combinators |  | | | |  | |  | | |  | |
| *Emotional exhaustion by* | | |  | | |  | | |  | | |
| SMBQ1 | | 0.986 | | 0.078 | | 0.000 | | 0.838 | | | 1.146 |
| SMBQ2 | | 1.202 | | 0.078 | | 0.000 | | 1.055 | | | 1.363 |
| SMBQ3 | | 1.448 | | 0.086 | | 0.000 | | 1.287 | | | 1.624 |
| SMBQ4 | | 1.115 | | 0.073 | | 0.000 | | 0.978 | | | 1.266 |
| SMBQ5 | | 1.343 | | 0.083 | | 0.000 | | 1.187 | | | 1.514 |
| SMBQ6 | | 0.874 | | 0.074 | | 0.000 | | 0.734 | | | 1.026 |
| *SMBQ1 with* |  | | | |  | |  | | |  | |
| SMBQ2 | | -0.059 | | 0.037 | | 0.055 | | -0.131 | | | 0.014 |
| SMBQ3 | | 0.008 | | 0.028 | | 0.385 | | -0.046 | | | 0.063 |
| SMBQ4 | | -0.010 | | 0.031 | | 0.377 | | -0.071 | | | 0.052 |
| SMBQ5 | | 0.007 | | 0.032 | | 0.418 | | -0.056 | | | 0.070 |
| SMBQ6 | | 0.094 | | 0.047 | | 0.023 | | 0.002 | | | 0.187 |
| *SMBQ2 with* |  | | | |  | |  | | |  | |
| SMBQ3 | | 0.030 | | 0.022 | | 0.089 | | -0.013 | | | 0.074 |
| SMBQ4 | | -0.005 | | 0.025 | | 0.423 | | -0.053 | | | 0.044 |
| SMBQ5 | | 0.000 | | 0.025 | | 0.498 | | -0.049 | | | 0.051 |
| SMBQ6 | | -0.050 | | 0.037 | | 0.089 | | -0.123 | | | 0.023 |
| *SMBQ3 with* |  | | | |  | |  | | |  | |
| SMBQ4 | | -0.010 | | 0.018 | | 0.284 | | -0.046 | | | 0.026 |
| SMBQ5 | | -0.006 | | 0.019 | | 0.381 | | -0.042 | | | 0.031 |
| SMBQ6 | | -0.017 | | 0.028 | | 0.270 | | -0.071 | | | 0.038 |
| *SMBQ4 with* |  | | | |  | |  | | |  | |
| SMBQ5 | | 0.012 | | 0.022 | | 0.293 | | -0.030 | | | 0.055 |
| SMBQ6 | | 0.041 | | 0.032 | | 0.094 | | -0.020 | | | 0.104 |
| *SMBQ5 with* |  | | | |  | |  | | |  | |
| SMBQ6 | | -0.004 | | 0.032 | | 0.448 | | -0.068 | | | 0.059 |

*Table 5. Sleep disturbances scalar model loadings and cross loadings.*

|  |  | | |  | |  | | | 95% CI | |
| --- | --- | --- | --- | --- | --- | --- | --- | --- | --- | --- |
|  | Estimate | | Posterior SD | | One-tailed p-value | | Lower 2.5% | | | Upper 2.5% |
| Employed and self-employed workers | | | | | | | | | | |
| Employed workers |  | | |  | |  | | |  | |
| *Sleep disturbances by* | |  | | |  | | |  | | |
| KSQ1 | 0.791 | | 0.035 | | 0.000 | | 0.722 | | | 0.861 |
| KSQ2 | 1.143 | | 0.035 | | 0.000 | | 1.076 | | | 1.213 |
| KSQ3 | 0.892 | | 0.038 | | 0.000 | | 0.819 | | | 0.967 |
| KSQ4 | 1.095 | | 0.037 | | 0.000 | | 1.024 | | | 1.169 |
| Self-employed workers | |  | | |  | | |  | | |
| *Sleep disturbances by* | |  | | |  | | |  | | |
| KSQ1 | 0.754 | | 0.053 | | 0.000 | | 0.653 | | | 0.861 |
| KSQ2 | 1.174 | | 0.072 | | 0.000 | | 1.035 | | | 1.318 |
| KSQ3 | 0.841 | | 0.057 | | 0.000 | | 0.731 | | | 0.956 |
| KSQ4 | 1.123 | | 0.070 | | 0.000 | | 0.989 | | | 1.262 |
| Employed workers and combinators | | | | | | | | | | |
|  |  | | |  | |  | | | 95% CI | |
|  | Estimate | | Posterior SD | | One-tailed p-value | | Lower 2.5% | | | Upper 2.5% |
| Employed workers |  | | |  | |  | | |  | |
| Sl*eep disturbances by* | |  | | |  | | |  | | |
| KSQ1 | 0.707 | | 0.057 | | 0.000 | | 0.599 | | | 0.822 |
| KSQ2 | 1.088 | | 0.063 | | 0.000 | | 0.970 | | | 1.216 |
| KSQ3 | 0.907 | | 0.062 | | 0.000 | | 0.790 | | | 1.031 |
| KSQ4 | 1.068 | | 0.060 | | 0.000 | | 0.954 | | | 1.191 |
| Combinators |  | | |  | |  | | |  | |
| *Sleep disturbances by* | |  | | |  | | |  | | |
| KSQ1 | 0.733 | | 0.070 | | 0.000 | | 0.601 | | | 0.876 |
| KSQ2 | 1.067 | | 0.086 | | 0.000 | | 0.905 | | | 1.242 |
| KSQ3 | 0.876 | | 0.077 | | 0.000 | | 0.732 | | | 1.033 |
| KSQ4 | 1.090 | | 0.086 | | 0.000 | | 0.926 | | | 1.265 |
| Self-employed workers and combinators | | | | | | | | | | |
|  |  | | |  | |  | | | 95% CI | |
|  | Estimate | | Posterior SD | | One-tailed p-value | | Lower 2.5% | | | Upper 2.5% |
| Employed workers |  | | |  | |  | | |  | |
| *Sleep disturbances by* | |  | | |  | | |  | | |
| KSQ1 | 0.784 | | 0.057 | | 0.000 | | 0.675 | | | 0.899 |
| KSQ2 | 1.044 | | 0.058 | | 0.000 | | 0.933 | | | 1.162 |
| KSQ3 | 0.788 | | 0.064 | | 0.000 | | 0.666 | | | 0.916 |
| KSQ4 | 0.973 | | 0.064 | | 0.000 | | 0.851 | | | 1.102 |
| Combinators |  | | |  | |  | | |  | |
| *Sleep disturbances by* | |  | | |  | | |  | | |
| KSQ1 | 0.726 | | 0.071 | | 0.000 | | 0.593 | | | 0.871 |
| KSQ2 | 1.009 | | 0.084 | | 0.000 | | 0.851 | | | 1.181 |
| KSQ3 | 0.810 | | 0.074 | | 0.000 | | 0.671 | | | 0.962 |
| KSQ4 | 1.023 | | 0.085 | | 0.000 | | 0.863 | | | 1.195 |

**
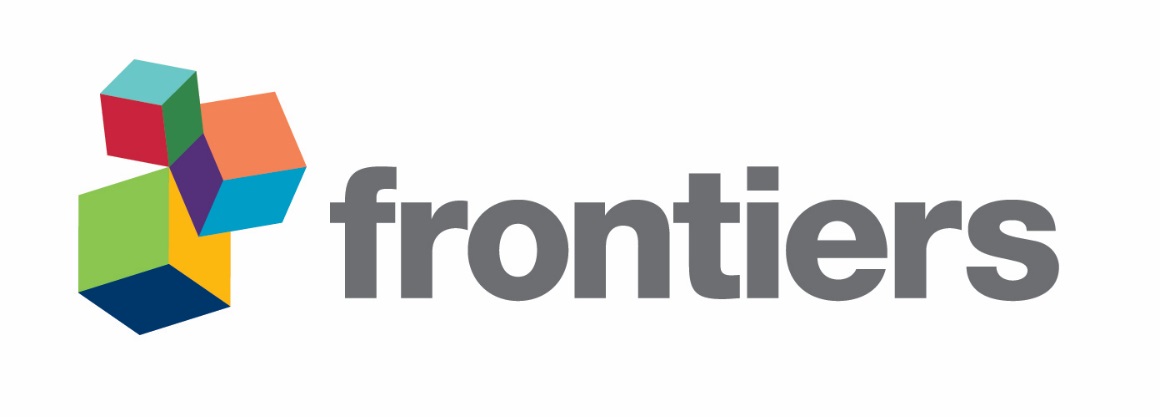
**
